# Moniliformediquinone as a potential therapeutic agent, inactivation of hepatic stellate cell and inhibition of liver fibrosis in vivo

**DOI:** 10.1186/s12967-016-1022-6

**Published:** 2016-09-09

**Authors:** Tsui-Hwa Tseng, Wea-Lung Lin, Zi-Hui Chen, Yean-Jang Lee, Ming-Shiun Shie, Kam-Fai Lee, Chien-Heng Shen, Hsing-Chun Kuo

**Affiliations:** 1 Department of Medical Applied Chemistry, Chung Shan Medical University, Taichung, Taiwan; 2 Department of Medical Education, Chung Shan Medical University Hospital, Taichung, Taiwan; 3 Department of Pathology, Chung Shan Medical University Hospital, Taichung, Taiwan; 4 Department of Chemistry, National Changhua University of Education, Changhua, Taiwan; 5 High Quality Biomedical Management & Consultant Inc., Taichung, Taiwan; 6 Department of Pathology, Chang Gung Memorial Hospital, Chiayi, Taiwan; 7 Graduate Institute of Clinical Medical Sciences, College of Medicine, Chang Gung University, Taoyuan, Taiwan; 8 Department of Hepato-Gastroenterological, Chang Gung Memorial Hospital, Chiayi, Taiwan; 9 Institute of Nursing and Department of Nursing, Chang Gung Institute of Technology, Chia-Yi Campus, Chiayi, Taiwan; 10 Chronic Diseases and Health Promotion Research Center, CGUST, Chiayi, Taiwan

**Keywords:** Moniliformediquinone, Hepatic fibrosis, CCl_4_-treated mice

## Abstract

**Background:**

Moniliformediquinone (MFD), a phenanthradiquinone in *Dendrobium moniliforme,* was synthesized in our laboratory. Beyond its in vitro inhibitory effects on cancer cells, other biological activity of MFD is unknown. The purpose of the present study was to investigate the effects of MFD on hepatic fibrogenesis in vitro and in vivo.

**Methods:**

Hepatic stellate HSC-T6 was cultured. Cell viability assay and western blot analyses were performed. Male ICR mice were evaluated on CCl_4_-induced hepatotoxicity using both histological examination and immunohistochemical staining.

**Results:**

First, in vitro study showed that the synthesized MFD effectively attenuated the expression of transforming growth factor-β1 (TGF-β1), connective tissue growth factor (CTGF), α-smooth muscle actin (α-SMA), and type I collagen (COL-1), which modulated the hepatic fibrogenesis. Furthermore, MFD reduced the phosphorylation of p65 NFκB in HSC-T6 cells. In vivo, liver fibrosis was induced by CCl_4_ twice a week for 10 weeks in mice. The administration of the MFD was started after 1 week of CCl_4_ thrice-weekly; the MFD significantly reduced plasma aspartate transaminase (AST) and lactose dehydrogenase (LDH) as well as hepatic hydroxy-proline, α-SMA, and COL-1 expression in CCl_4_-treated mice. Pathological analysis showed that the MFD alleviated CCl_4_-induced hepatic inflammation, necrosis and fibrosis. These results suggest that MFD possesses therapeutic potential for liver fibrosis.

**Conclusions:**

The synthesized MFD exhibits anti-fibrotic potential by inactivation of HSCs in vitro and decreases mouse hepatic fibrosis in vivo. Further investigation into their clinical therapeutic potential is required.

## Background

Liver cancer is usually an aggressive malignant disease with a poor prognosis. Although recent advances have been made in the diagnosis and treatment of tumors, the development of clinical metastasis remains a significant cause of mortality from this disease. Accordingly, hepatic fibrosis represents the final pathological outcome for the majority of chronic liver insults [1–3]. Untreated fibrosis may progress to liver cirrhosis, ultimately leading to organ failure, hepatoma and death [4, 5]. Unfortunately, effective clinical therapies are still lacking. Liver fibrosis is a progressive pathological process as part of the wound healing and tissue remodeling mechanism in response to chronic liver insults ranging from viral infections to various toxins [5, 6]. During liver fibrosis, excessive extracellular matrix (ECM) is produced and accumulated, leading to liver dysfunction and irreversible cirrhosis [7, 8]. Hepatic stellate cells (HSCs) have been identified as the principle cellular source of this ECM, even though several other cell types were also recruited to the damaged lesions and differentiated to fibroblastic cells. After acute liver injury and in chronic liver disease, quiescent HSCs become activated and transdifferentiate into myofibroblast-like cells characterized by several key phenotypic changes, such as producing excessive extracellular matrix (ECM) including type I collagen (COL-1) and α-smooth muscle actin (α-SMA). Activated HSCs perpetuate their own activation through several autocrine loops, including the secretion of transforming growth factor-β1 (TGF-β1) and connective tissue growth factor (CTGF) [9]. Therefore, the modulation function of activated HSCs has been proposed as a therapeutic strategy against hepatic fibrosis, for instance, decreasing proliferation of HSCs and reducing production of ECM and cytokines.

**Fig. 1:**
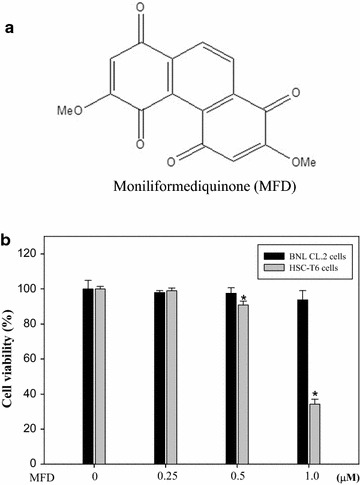
**a** Chemical structure of moniliformediquinone (MFD). **b** Effect of MFD on cell viability of HSC-T6 cells. HSC-T6 cells were treated with MFD (0–2.5 μM) for 24 and 48 h. MTT assays were performed to assess cell viability. The data represent mean ± SD of three independent experiments. *P < 0.01 versus the control

Accordingly, hepatotoxins cause liver damage characterized by varying degrees of hepatocyte degeneration and cell death, such as ethanol, acetaminophen, and carbon tetrachloride (CCl_4_). For instance, CCl_4_ impairs hepatocytes directly by altering the permeability of the plasma, lysosomal, and mitochondrial membranes. In addition, CCl_4_-induced liver fibrosis shows many characteristics with human fibrosis of different etiologies [10]. Furthermore, CCl_4_-induced hepatic injury has been extensively used in animal models to evaluate the therapeutic potential of drugs. On the other hand, plants of the *Dendrobium* genus (*Orchidaceae*) are used in traditional herbal medicines in Asia, and studies have shown that they contain a wide variety of medicinal properties [11–13]. Moniliformediquinone (2,6-dimethoxy-1,4,5,8-phenanthradiquinone; MFD) (Fig. 1), collected by Chang et al. has been isolated from stems of *Dendrobium monilifore* (L.) Sw. in Taiwan and has been found to have anti-inflammation and anticancer effects [14]. In addition, our previous study of synthesized MFD showed that synthesized MFD could induce in vitro and in vivo antitumor activity through a glutathione-involved DNA damage response and mitochondrial stress in human hormone refractory prostate cancer [15]. Other biological activities of MFD are unknown. In the present study, the anti-hepatic fibrosis potential of synthesized MFD was evaluated using HSC-T6, an activated HSC cell line, and a mouse model of CCl_4_-induced hepatic injury. Further study aimed to investigate the effect of synthesized MFD on the expression of activated HSC-T6 markers and fibrosis-related proteins and its relationship with the expression of TGF-β1, α-SMA, and COL-1. Finally, pathological analysis showed that the synthesized MFD could possess therapeutic potential for liver fibrosis.

## Methods

### Chemicals

Dulbecco’s modified Eagle’s medium (DMEM), phosphate-buffered saline (PBS), fetal bovine serum, *l*-glutamine, penicillin/streptomycin (PS), and trypsin-EDTA were purchased from GIBCO Ltd. (Grand Island, NY, USA). Anti-CTGF, α-SMA, and α-tubulin antibodies were provided from Santa Cruz Biotechnology (Santa Cruz, CA). Anti-TGF-β1 was obtained from Cell signaling Technology (Beverly, MA). Other chemical reagents were purchased from Sigma-Aldrich.

The 2,6-dimethoxy-1,4,5,8-phenanthrenetetrone, moniliformediquinone (MFD), was prepared as previous procedure [16]. Melting point and ^1^H and ^13^C NMR spectra of the synthetic MFD are in agreement with those reported for the natural product [14]. Thus, the drug of synthetic MFD is >95 % purity (^13^C NMR peak purity test) for following bioassay. The TdT-mediated dUTP Nick End Labeling (TUNEL) kits were purchased from Roche (Germany).

### Cell culture and MTT assay

The immortalized hepatic stellate cell line (HSC-T6) and murine liver cell (BNL CL.2) were maintained in Dulbecco’s modified Eagle’s medium (DMEM) plus 10 % (v/v) fetal bovine serum, penicillin-streptomycin (100 IU/mL–100 μg/mL), and incubated at 37 °C in a 5 % CO_2_ humidified atmosphere. Cell viability was determined using MTT assay. In brief, cells (2–5 × 10^4^ cells/well) were seeded in 24-well culture plates and exposed to different concentrations of moniliformediquinone for 24 h. Next, the medium was changed and the cells were incubated with MTT (5 mg/mL) for 4 h. Finally, the absorbance of the formazan product was measured at a wavelength of 570 nm on an ELISA reader [17].

### Preparation of total cell extracts and immunoblots analysis

Cells (1 × 10^6^ cells/well) were seeded in 10-cm dishes in the presence of the MFD. The cells were collected by trypsin-EDTA and lysed in RIPA buffer (50 mM Tris-HCl, 1 mM EDTA, 150 mM NaCl, 1 % NP-40) containing protease inhibitors. After mixing for 30 min at 4 °C, the mixtures were centrifuged (10,000×*g*) for 10 min at 4 °C and the supernatants were collected as whole-cell extracts. The protein content was determined using the Bio-Rad protein assay reagent and bovine serum albumin as a standard. An equal amount of protein from the total cell extracts was boiled for 8 min. The extracts were separated by SDS-polyacrylamide gels and transferred to a NC membrane (Whatman). The blots were blocked in 5 % non-fat dry milk/PBS for 1 h at room temperature. The blots then were incubated overnight with primary antibodies, followed by horseradish peroxidase-conjugated goat anti-mouse (or rabbit) IgG for 1 h. The immunoreactive bands were revealed by enhanced chemiluminescence with a commercially available ECL kit [18].

### Animals

Male ICR mice (body weight 18–22 g) were purchased from GlycoNex Inc. (Taiwan) and maintained in cage housing in a specifically designed pathogen-free isolation facility with a 12/12 h light/dark cycle. Animal care and the general protocols for animal use were approved by the Institutional Animal Care and Use Committee of Chung Shan Medical University Animal Ethics Research Board. All mice of CCl_4_-treated alone and combined MFD administration group were given 0.1 mL/mice of CCl_4_ (20 % CCl_4_ in olive oil) via intragastric twice a week for 10 weeks. The mice were intraperitoneal injected with 0.1 and 0.5 mg/kg of MFD thrice a week. Those in control and MFD-treated alone groups were given equal volume of olive oil. The mice were divided randomly into five groups of eight mice each. Then, the mice were sacrificed 24 h after the last injection. The livers were divided into two portions (1) preserved in 10 % formalin for histological examination, (2) frozen for immunoblotting analysis at −70 °C.

### Biochemical assays

Blood was obtained by intra-cardiac puncture from mice. Serum was separated and levels of alanine aminotransferase (AST) and lactate dehydrogenase (LDH) were measured using standard enzymatic assay kits. Each assay is a colorimetric assay with detection of a highly colored end product measured at 490–520 nm. The absorbance of each end product is proportional to the enzyme’s activity [19].

Liver samples were lysed in radioimmunoprecipitation assay (RIPA) buffer containing 50 mM Tris-HC pH 7.4, 150 mM NaCl, 1 % NP-40, 0.5 % sodium deoxycholate, 0.1 % SDS, 2 mM phenylmethylsulfonyl fluoride, 1 mM sodium orthovanadate, and 2 μg/mL of each leupeptin and pepstatin. Volume equivalent to 50 μg of proteins was analyzed by western immunoblotting assay as previous description.

### Histopathological analysis

Liver tissues from each mouse were rapidly removed, fixed in 10 % neutral-buffered formalin, and processed routinely. Paraffin-embedded sections were cut into 4 μm thick sections. The sections were stained with hematoxylin and eosin (H&E) and with Masson’s trichome for collagen fibers [20].

### Immunohistochemistry

Immunohistochemistry (IHC) staining was performed using a biotinylated secondary antibody (Vectastain Universal Elite ABC Kit, Burlingame, CA, USA). Monoclonal rabbit antibodies against peroxiredoxin 2 and alpha-1-antiproteinase were diluted at a ratio of 1:100. The omission of primary antibodies was used as the negative control. For three slides, cytoplasm that was stained brown was scored as positive. The expression of hydroxy-proline, α-SMA and COL-1 were quantitatively evaluated using an Olympus CX31 microscope (Tokyo, Japan) with the Image-pro Plus medical image analysis system. Digital images were captured using a digital camera (Canon A640, Tokyo, Japan). The positive area and optical density (OD) of hydroxy-proline, α-SMA and COL-1 positive cells were determined by measuring three randomly selected microscopic fields (400× magnification) for each slide. The IHC index was defined as the average integral optical density (AIOD) (AIOD = positive area × OD/total area) [21].

### Statistical analysis

All experiments were carried out in triplicate, and data were expressed as mean ± SD. One-way analysis of variance (ANOVA) and the Duncan test were carried out to determine significant differences of multiple comparisons. Significance was defined as a *P* value of less than 0.05 [19].

## Results

### Cytotoxicity of MFD in HSC-T6 and BNL CL.2 cells

Figure 1 shows the effects of various concentrations of MFD on activated HSCs (HSC-T6) and normal murine liver cells (BNL CL.2). After treatment for 48 h. MFD showed a cytotoxicity effect on HSC-T6 cells above 0.5 μM concentrations whereas the applied concentration showed noncytotoxicity on BNL CL.2 cells.

### Effect of MFD on the expression of TGF-β, CTGF, α-SMA, COL-1 in HSC-T6 cells

**Fig. 2:**
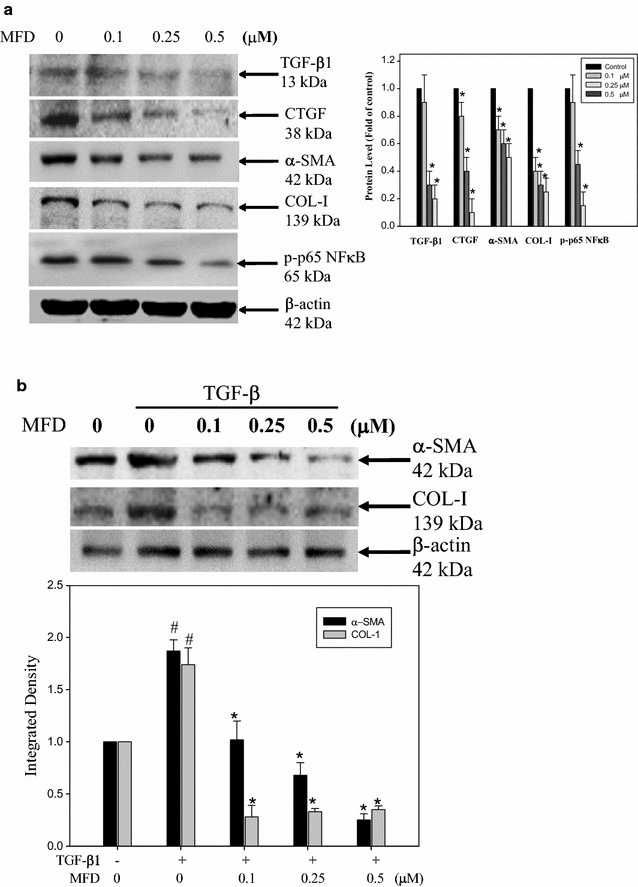
Effect of MFD on the expression of activated HSC-T6 markers and fibrosis-related proteins. HSC-T6 cells were treated with various concentrations of MFD for 48 h; then total cell lysates were tested for (**a**) TGF-β1, CTGF, α-SMA, COL-1 and p-p65 NFκB by western blot analysis. **b** HSC-T6 cells were treated with TGF-β1 (2 ng/mL) alone or in combination with different concentrations of MFD for 48 h; then total cell lysates were analyzed by western blot analysis against anti-CTGF, anti-α-SMA, anti-COL-1, and anti-β-actin as an internal control. Quantification of band intensity relative to α-tubulin is shown below the western blot. The quantitative data were presented as the mean of three repeats from one independent experiment. The data were presented as mean ± SD of three independent experiments. ^#^ *P* < 0.05 versus control. *P < 0.05, versus TGF-β1-treated alone

Transforming growth factor-β1 (TGF-β1) is a major fibrotic growth factor in liver fibrosis [9]. CTGF is selectively induced by TGF-β in a fibroblastic cell type and plays a key role in the overproduction of ECM in activated HSCs. Therefore, we determined the levels of TGF-β1 and CTGF after treatment with MFD in HSC-T6 cells using western blot analysis. The results showed that MFD significantly decreased the protein level of TGF-β1 and CTGF (Fig. 2a). In addition, the most striking biological consequences of activated HSCs are the marked accumulation of α-SMA and the dramatic increase in collagen depositions. MFD reduced the levels of α-SMA and COL-1 after treatment with MFD in HSC-T6 cells as compared with the control (Fig. 2a). Furthermore, MFD inhibited the phosphorylation of p65 NFkB (Fig. 2a), which has been suggested to be associated with the regulation of TGF-β1 expression [22]. To confirm the important role of TGF-β1 affected by MFD, TGF-β1 was added to promote the expression of CTGF, α-SMA, and COL-1. As shown in Fig. 2b, MFD reduced the expression of CTGF, α-SMA, and COL-1, which were mediated by TGF-β1.

### Inhibitory effect of MFD on CCl_4_-induced hepatotoxicity in mice

**Fig. 3:**
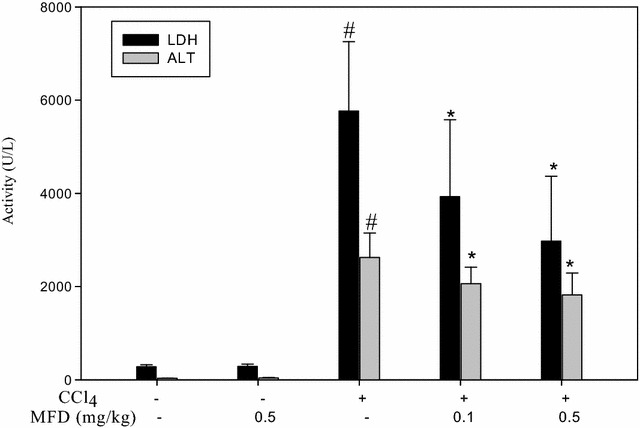
Effects of MFD on serum AST and LDH in CCl_4_-treated mice. The data for serum LDH and AST are presented as mean ± SD from six mice per group. ^#^ *P* < 0.05, as compared to the control group; *P < 0.05, as compared to the CCl_4_-treated group

In order to evaluate hepatic tissue damage, serum enzyme levels of LDH and AST with/without CCl_4_-treated mice were determined by using standard enzymatic kits. As shown in Fig. 3, serum LDH and AST activity in mice treated with MFD alone did not significantly differ from that of the control group. AST and LDH activity of all CCl_4_-treated mice increased significantly as compared with that of the control group after the end of the experiment (*P* < *0.001*). However, administration of the MFD significantly suppressed the CCl_4_-induced increase of LDH and AST activity, respectively (*P* < *0.01*, *P* < *0.001*).

### MFD inhibits CCl_4_-induced TGF-β1, α-SMA and COL-1 expression in mouse liver

**Fig. 4:**
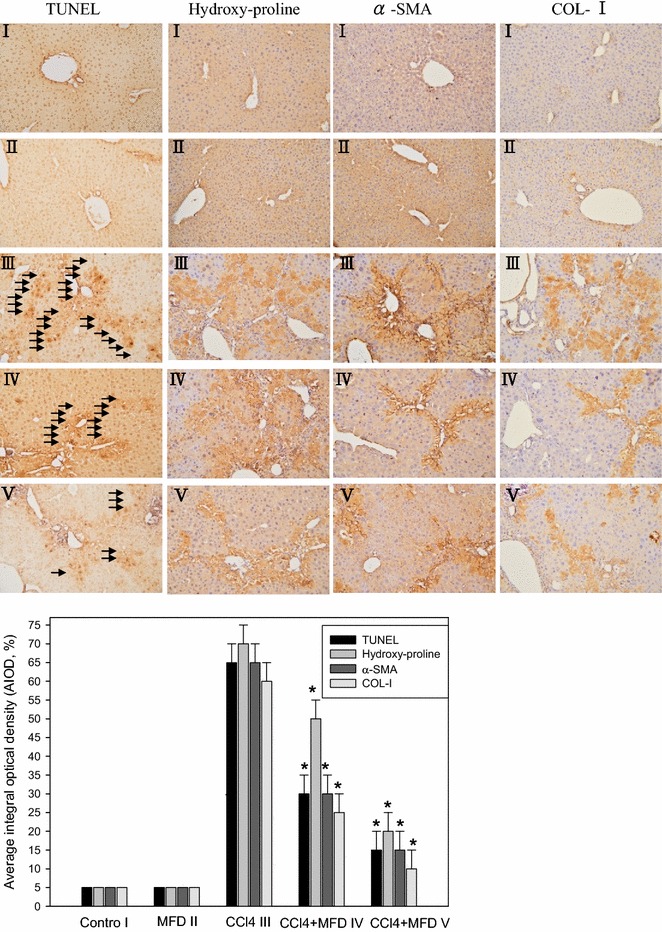
Effect of MFD on the liver fibrosis-related protein expression in CCl_4_-treated mouse liver. Histological examination of liver were revealed as indicated by immunohistochemical staining of TUNEL. Hydroxy-proline, α-SMA and COL-1 were evaluated by immunohistochemical staining. Representative liver sections stained as oil infusion control group (*I*); Mice with MFD treatment (*II*); Mice with CCl_4_ injection (*III*); MFD treatment (0.1 and 0.5 mg/kg) (*IV*, *V*); Magnification, ×200. The cells were counted from 10 fields (×200 magnification) of each liver sample. The results from statistical analysis are the means of cells and were calculated per microscope field from eight animals per group. Data are expressed as mean ± SD of independent experiments. *P < 0.05, as compared to the CCl_4_-treated group

The TUNEL assay results are shown in Fig. 4. Global immunoreactivity to apoptosis was localized primarily within liver tissue in CCl_4_ mouse. Quantitative examination of hepatocyte pathology showed that the number of normal hepatocytes present in the CCl_4_ treatment group was lower than the number of normal hepatocytes present in the control groups (control group = 5 ± 1; CCl_4_ treatment group = 65 ± 3; 0.1 mg/kg MFD treated CCl_4_ = 30 ± 3; 0.5 mg/kg MFD treated CCl_4_ = 15 ± 3, *P* < 0.05). We employed immunohistochemistry to examine liver hydroxy-proline protein expression in the hepatocyte of the untreated control and MFD alone group, the CCl_4_ group and the two CCl_4_ + MFD groups, which acts as an index of oxidative damage in hepatic fibrosis, was measured in order to investigate the therapeutic effect of MFD. A significant reduction in hydroxy-proline was found in the CCl_4_ + MFD groups compared to the CCl_4_ group. Significantly decreased expression of α-SMA, and COL-1 proteins were found in the MFD groups compared to the CCl_4_ group, *P* < 0.05 (Fig. 4).

### Histopathological examination

**Fig. 5:**
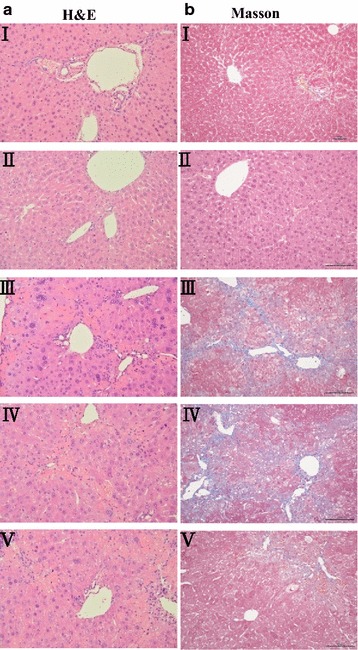
Effect of MFD on histological changes in the liver of CCl_4_-treated mice. Lliver sections stained as oil infusion control group (*I*); mice with MFD treatment (*II*); mice with CCl_4_ injection (*III*); MFD treatment (0.1 and 0.5 mg/kg) (*IV*, *V*); the section of mouse liver was stained with **a** hematoxylin-eosin or **b** Masson’s trichrome (magnification ×200)

As shown in Fig. 5, in vehicle control and MFD-only-treated mice, liver sections showed normal hepatic cells, i.e., with a well-preserved cytoplasm, a prominent nucleus, and a central vein. The livers of CCl_4_-intoxicated mice revealed moderate to severe hepatocellular vacuolization, hepatic necrosis, and inflammatory cell filtration. Against the pathological changes, the lesions of the 0.5 mg/kg MFD-treated mice were of a much milder degree compared with the lesions observed in the group treated with CCl_4_ alone. In addition, the collagen of fibrotic tissues showed a blue color when stained by Masson’s trichrome. There was no fibrosis in either the control group or the group treated with MFD alone. In contrast, a large number of blue collagen fibers were observed in the CCl_4_-treated mouse (Fig. 5). In the 0.5 mg/kg MFD-treated group, the extent and area of the collagen fibers was apparently reduced (Table 1).

**Table 1:** MFD exhibits anti-fibrotic potential by decreasing mouse hepatic fibrosis in vivo

	Inflammation	Degeneration	Hepatocyte necrosis	Fibrosis
Control	−	−	−	−
MFD alone	−		−	−
CCl_4_ alone	+++	++	+++	+++
CCl_4_ + MFD (0.1 mg/kg)	+++	++	+++	++
CCl_4_ + MFD (0.5 mg/kg)	++	+	+	+

## Discussion

Hepatic stellate cells (HSC) are considered to play a key role in the pathogenesis of liver fibrosis. During liver fibrogenesis, HSCs are activated and acquire a myofibroblast-like phenotype that is accompanied by increased proliferation and extracellular matrix synthesis [23, 24]. Therefore, suppression of HSC activation has been proposed as a therapeutic target against hepatic fibrosis. In the present study, synthesized MFD was measured for its anti-fibrotic activities by assessing the effect on the suppression expression of fibrotic-associated protein. α-SMA is a definite marker of transdifferentiation of stellate cells. The experimental data showed that MFD reduced α-SMA expression in HSC-T6 cells and in the liver of the CCl_4_-treated mouse (Figs. 2, 4). Furthermore, collagens such as COL-1 are the main components of the ECM, and the main collagen-producing cells in the liver are HSCs. The present study found that MFD decreased COL-1 expression in HSC-T6 cells and in the liver of the CCl_4_-treated mouse (Figs. 4, 5). These results showed that MFD can inhibit HSCs activation. In addition, it first demonstrated, in vitro and in vivo, an anti-fibrotic effect of MFD, which has been isolated from the *Dendrobium monilifore* stem. Moreover, phenanthrenes from the *Dendrobium nobile* stem have previously been shown to exhibit anti-fibrotic potential [13]. Consistent with these findings, the *Dendrobium* species is thought to be useful for developing therapeutic agents for the treatment of hepatic fibrosis.

Many studies have evaluated candidate anti-fibrosis factors or reagents by assessing their impact on TGF-β-induced collagen and α-SMA expression in fibroblasts. For instance, TGF-β1 is a pleiotropic cytokine involved in the activation of HSCs, the main producers of ECM components in the liver [25]. In general, TGF-β1 induces the expression of type I collagen and inhibits apoptosis of HSCs. It is also suggested that TGF-β1 can up-regulate the platelet-derived growth factor beta (PDGF-β) receptor, resulting in a proliferation response in activated HSCs [26]. As shown in Fig. 3, MFD inhibited DNA synthesis in correlation with reducing the expression of TGF-β1. There are many regulators involved in the inflammation-fibrosis process, and the NFκB signaling pathway appears to play a critical role in liver homeostasis, pathophysiology, and regulation of inflammation-fibrosis [27]. NFκB is found in activated HSC [28], where it is involved in upregulating proinflammatory gene encoding adhesion molecules, chemokines, and cytokines such as TGF-β1. Our study found that MFD inhibited the phosphorylation of p65 NFκB and the expression of TGF-β1 (Fig. 2a, b). NFκB was also involved in the protection of HSC from apoptosis. Suppression of NFκB may increase apoptosis of HSC. As shown in Fig. 2b, MFD can reduce the TGF-β1-stimulating expression of α-SMA, COL-1, and CTGF, which are downstream of TGF-β1. These data imply that MFD possesses an anti-fibrogenesis effect by inhibiting HSCs activation associated with blocking the NFκB signaling pathway and reducing TGF-β1 expression. The molecular mechanism of the signaling pathway regulated by MFD in HSC needs further clarification.

Hepatic fibrosis, a leading cause of morbidity and mortality worldwide, is usually associated with chronic liver disease. Hepatic fibrosis can develop into cirrhosis within 1–10 years [29]. Thereafter, blocking the progression of fibrosis may be an efficacious strategy to survival. As a consequence, CCl_4_-induced hepatic injury is an experimental model used for drug screening [30]. In addition, AST and LDH are enzymatic indicators of tissue damage by toxicants or disease condition, and abnormal levels of AST and LDH are important to pathology and toxicology. According to our in vivo study, administration of MFD significantly reduced the level of AST and LDH as compared with the group treated with CCl_4_ alone (Fig. 3). In addition, MFD inhibited CCl_4_-induced hepatic fibrotic factor expression such as hydroxy-proline, α-SMA, and COL-1 (Fig. 4). Histopathological examination showed that 0.5 mg/kg MFD apparently decreased CCl_4_-induced hepatic inflammation, necrosis, and fibrosis (Fig. 5). These results demonstrated that the synthesized MFD can block CCl_4_-induced hepatic damage and fibrotic progression. Based on our results, it makes possible that cultured HSC underwent a rapid and persistent induction NF-kB, while the inhibition of NF-kB as well as CTGF, α-SMA and COLI by MFD were required in a dose-dependent. In this study, we investigated MFD has an inhibitory effect on the CCl_4_ model of hepatocyte injury that is cytotoxic to serious fibrotic liver in vivo. Further investigation is required to better understand whether when CCl_4_ infusion in rats were established as model of liver dysfunction and fibrogenesis, treatment with MFD induced a significant dose-dependent decrease in liver fibrosis for a period of time points. These results provide insights into the therapeutic activity of MFD, which thus may be promising candidates for the treatment of liver injury diseases such as hepatic fibrosis.

## Conclusions

In summary, this study concludes that the synthesized MFD exhibits anti-fibrotic potential by inactivation of HSCs in vitro and decreases mouse hepatic fibrosis in vivo. Further investigation into their clinical application is required.

## Abbreviations

HSCs: hepatic stellate cells
BSA: bovine serum albumin
DMSO: dimethylsulfoxide
FBS: fetal bovine serum
ECM: extracellular matrix
CTGF: connective tissue growth factor
α-SMA: α-smooth muscle actin
COL-1: type I collagen
NF-κB: nuclear factor-κB
PBS: phosphate buffered saline
TGF-β: transforming growth factor-β
AST: aspartate transaminase
LDH: lactose dehydrogenase
